# Glycemic Control after Total Pancreatectomy for Intraductal Papillary Mucinous Neoplasm: An Exploratory Study

**DOI:** 10.1155/2012/381328

**Published:** 2012-08-26

**Authors:** Laith H. Jamil, Ana M. Chindris, Kanwar R. S. Gill, Daniela Scimeca, John A. Stauffer, Michael G. Heckman, Shon E. Meek, Justin H. Nguyen, Horacio J. Asbun, Massimo Raimondo, Timothy A. Woodward, Michael B. Wallace

**Affiliations:** ^1^Division of Gastroenterology and Hepatology, Mayo Clinic, Jacksonville, FL 32224, USA; ^2^Division of Endocrinology, Mayo Clinic, Jacksonville, FL 32224, USA; ^3^Department of Surgery, Mayo Clinic, Jacksonville, FL 32224, USA; ^4^Biostatistics Unit, Mayo Clinic, Jacksonville, FL 32224, USA; ^5^Department of Transplantation, Mayo Clinic, Jacksonville, FL 32224, USA

## Abstract

*Background*. Glycemic control following total pancreatectomy (TP) has been thought to be difficult to manage. Diffuse intraductal papillary mucinous neoplasm (IPMN) is a potentially curable precursor to pancreatic adenocarcinoma, best treated by TP. *Objective*. Compare glycemic control in patients undergoing TP for IPMN to patients with type 1 diabetes mellitus (DM). *Design/Setting*. Retrospective cohort. *Outcome Measure*. Hemoglobin A1C(HbA1C) at 6, 12, 18, and 24 months after TP. In the control group, baseline was defined as 6 months prior to the first HbA1c measure. *Results*. Mean HgbA1C at each point of interest was similar between TP and type I DM patients (6 months (7.5% versus 7.7%, *P* = 0.52), 12 months (7.3% versus 8.0%, *P* = 0.081), 18 months (7.7% and 7.6%, *P* = 0.64), and at 24 months (7.3% versus 7.8%, *P* = 0.10)). Seven TP patients (50%) experienced a hypoglycemic event compared to 65 type 1 DM patients (65%, *P* = 0.38). *Limitations*. Small number of TP patients, retrospective design, lack of long-termfollowup. *Conclusion*. This suggests that glycemic control following TP for IPMNcan be well managed, similar to type 1 DM patients. Fear of DM following TP for IPMN should not preclude surgery when TP is indicated.

## 1. Introduction

Diabetes mellitus (DM) induced by total pancreatectomy (TP), often termed “Pancreatogenic Diabetes,” is often thought to be difficult to manage [1–4]. The notion that TP could cause brittle diabetes in up to 25% of patients may adversely influence the decision to perform the surgery. In addition, the overall quality of life will likely be affected by such intervention [5]. More recent data suggests that glycemic control following TP may not be as challenging as initially thought [6]. Intraductal papillary mucinous neoplasm (IPMN) is a distinct pathological entity comprised of a papillary proliferation of mucin-producing epithelial cells that may produce excessive mucus and may cause cystic dilation of the pancreatic duct [7]. IPMN has a broad histological spectrum, ranging from minimal mucinous hyperplasia or adenoma to invasive carcinoma [8]. Criteria for pancreatic resections in IPMN, including TP, have been proposed [9]. IPMN involvement of the main pancreatic duct has been shown to be a risk factor for prevalent and incident cancers and therefore is a leading cause for recommending surgical resection [8]. Recent evidence reports that TP for IPMN is gaining popularity [10–14].

Many published studies evaluating glycemic control post-TP have included all patients undergoing TP regardless of etiology [5, 6]. Blanchet et al. reported a series of 10 patients in which glycemic control was achieved successfully after TP for mucinous pancreatic tumors; seven of those patients had IPMN [15].

 It is unknown whether the underlying pancreatic disease has any impact on insulin production prior to TP, which may affect glycemic control after surgery.

 Most studies evaluating glycemic control in these patients were performed prior to the availability of more advanced treatment modalities of DM such as insulin pumps [1–5].

The aim of this exploratory study was to evaluate glycemic control in patients undergoing TP for IPMN and compare them to a control group of patients with type 1 DM, who were being followed during the same period. This included both long-term control, through measuring HbA1c, as well as occurrence of reported glycemic control-related complications such as hypoglycemia and hyperglycemia. We also evaluated the outcome of these reported episodes. This data stemmed and was expanded from a previously published study where we examined the outcome of TP for various indications [16].

## 2. Methods

We performed a retrospective chart review of all patients who underwent TP for IPMN between July 2004 and July 2008 at Mayo Clinic, in Jacksonville, Florida. We identified 29 patients. Follow-up data was available in 19 patients. Patients were included if they had at least one HbA1c measurement at any of the 4 time points of interest (6 [±3], 12 [±3], 18 [±3], or 24 [±3] months after TP). Such data was available for 14 of the 29 patients (48%). Sample sizes at each of the four time points were *N* = 10, *N* = 9, *N* = 7, and *N* = 6, respectively. The date of TP was considered as the baseline time point in TP patients. Of the 14 patients included in this study, 2 had type 2 DM prior to surgery. When comparing the 14 included TP patients with the 15 patients who were excluded due to insufficient data, no significant difference regarding age at surgery, gender, weight, BMI, pancreatic enzyme supplement use, or insulin regimen was noted (all *P* ≥ 0.11).

Type I DM patients were included if at baseline, which was defined as 6 months prior to the first HbA1c measure, their duration of disease was at least 2 years. HbA1c measures in controls were considered at the same four time points as the TP patients (6 [±3], 12 [±3], 18 [±3], or 24 [±3] months after baseline). We identified 366 patients with an ICD code corresponding to type 1 diabetes mellitus (medical record numbers in arithmetical order) from our outpatient clinic. We selected every 5th patient on the list; after that, we continued with every 5th patient from the remaining list and so on until we identified 100 patients that we used as controls. Patients who were found to have type 2 DM during chart review were excluded. These patients were treated in our clinic within the same timeframe as the IPMN patients, between July 2003 and July 2006 and therefore had access to the same therapeutic means as our patient population. All patients had HbA1C measured within the interval studied.

### 2.1. Total Pancreatectomy Insulin Regimens and Doses

All patients were started on an insulin infusion following surgery and were discharged on meal time insulin Aspart (Novo Nordisk, Bagsvaerd, DN) with a correction scale. In addition, patients were given either Recombinant Insulin Glargine (Sanofi-Aventis, Bridgewater, N.J.) (13 patients) or Insulin Detemir (Novo Nordisk, Bagsvaerd, DN) (one patient), based on the preference of their endocrinologist. Their most current insulin regimens were Recombinant Insulin Glargine2-24 units once a day along with Insulin Aspart per sliding scale for meal coverage in 10 patients, Insulin pump in 3 patients, and Insulin Detemir12 units in the morning and four units in the evening along with Insulin Aspart per sliding scale for meal coverage in 1 patient.

### 2.2. Statistical Analysis

Patient characteristics at baseline were compared between TP patients and type I DM patients using a Wilcoxon rank-sum test or Fisher's exact test. In the primary analysis, we compared mean HbA1c values between TP patients and type I DM patients using a two-sample *t*-test separately at each time point. We also estimated the difference in mean HbA1c between groups along with a 95% confidence interval (CI). Additionally, we examined the sensitivity of the results to the adjustment for potentially confounding variables in multivariable linear regression analysis, adjusting for any variable that differed significantly (*P* ≤ 0.05) between TP patients and type I DM patients. In secondary analysis, again separately at each time point, we estimated the proportion of patients with a HbA1c level of less than 7% for TP patients and type I diabetes patients using exact binomial 95% CI and compared these proportions using Fisher's exact test. We estimated the difference in this proportion between groups along with a 95% small sample CI using Newcombe's score method [17]. No adjustment for potentially confounding variables was made in this secondary analysis, owing to the limitations on the number of variables that can be reasonably adjusted for in a regression model involving a dichotomous outcome as opposed to a continuous outcome [18]. We also evaluated trends in HbA1c values over time, separately in TP and type I DM patients, using mixed effects linear regression models including a random effect for patient. *P*-values less than or equal to 0.05 were considered statistically significant. All statistical analyses were performed using SPLUS (version 8.0.1; Insightful Corporation, Seattle, Washington).

## 3. Results

### 3.1. Patient Characteristics

Patient characteristics at baseline for TP and type I DM patients are shown in Table 1. TP patients were older (median: 72 years versus 52 years, *P* < 0.001), while the control group had more men (52% versus 14%, *P* = 0.01), more years of education (median: 16 years versus 12 years, *P* = 0.034), and were heavier at baseline (median: 78 kg versus 60 kg, *P* = 0.028) when compared to type I DM patients. BMI was not significantly different between the two groups (median: 26 versus 24, *P* = 0.47). The median duration of disease in type I DM patients was 26 years (range: 2 years–55 years). The indication for TP was diffuse involvement of the pancreas in 11 patients and positive margins during surgery in the remaining three patients. On pathology, mucinous adenocarcinoma was noted in one patient, noninvasive carcinoma in eight patients, adenoma in four patients, and one patient had borderline findings for malignancy. None had lymphovascular invasion. There was no recurrence of disease noted in these patients during their followup.

### 3.2. Glycemic Control

Mean HbA1c was similar between TP and type I DM patients at six months (7.5% versus 7.7%, *P* = 0.74), 12 months (7.3% versus 8.0%, *P* = 0.11), 18 months (7.7% and 7.6%, *P* = 0.79), and at 24 months (7.3% versus 7.8%, *P* = 0.31) (Table 2). These findings remained consistent when adjusted for age at baseline, gender, weight at baseline, and years of education (Table 2), all of which differed significantly between groups. There was no evidence of a difference in HbA1c values between the 6-month, 12-month, 18-month, and 24-month time points in TP patients (*P* = 0.37) or type I DM patients (*P* = 0.46).

**Figure 1:**
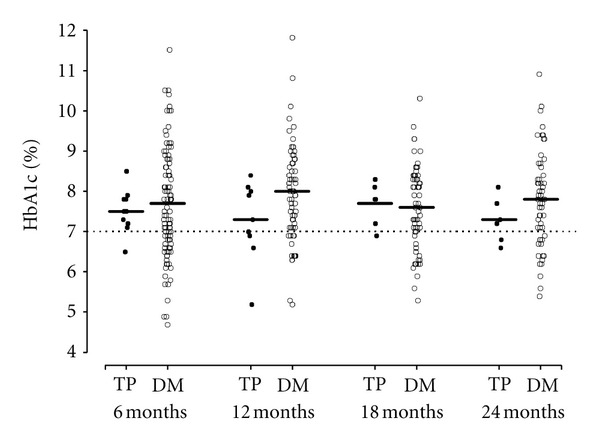
HbA1c values after baseline in TP and type I diabetes mellitus patients. The sample mean is shown with a solid horizontal line.

Differences in the proportion of patients with an HbA1c less than 7% at each time point of interest after baseline were also not significant (all *P* ≥ 0.42) between TP and control patients (10% versus 33% at 6 months, 33% versus 21% at 12 months, 14% versus 25% at 18 months, and 33% versus 28% at 24 months) (Table 3). The individual HbA1c values for TP patients and controls for the different time periods are shown in Figure 1.

### 3.3. Glycemic Control-Related Complications

When considering the presence of a symptomatic hypoglycemic event at any point during the study period after baseline, seven TP patients (50%) experienced a hypoglycemic episode compared to 65 type I DM patients (65%) (*P* = 0.38). Six out of seven TP patients (86%) who experienced a hypoglycemic episode treated the episode themselves at home, compared to 59 type I DM patients (91%). The remaining 6 type I DM patients (9%) received treatment at a hospital compared to 1 TP patient who required admission to the emergency room, where she was treated with intravenous Dextrose 50% and discharged home. No patient reported a hyperglycemic episode that required hospitalization or evaluation in the emergency department.

### 3.4. Pancreatic Insufficiency

Following hospital discharge, 13 of 14 TP patients continued on pancreatic enzyme supplements to avoid malabsorption, with its potential negative effects on glycemic control. Only two patients continued to complain of steatorrhea because of intolerance of medications (one patient) and inadequate dosing (one patient).

## 4. Discussion

The findings of our exploratory study suggest that glycemic control following TP may be manageable, with control and complication rates similar to that of typical type 1 DM patients who have not undergone pancreatectomy. Our focus on IPMN patients offered a more homogenous patient population with a relatively reduced list of comorbidities that could influence the results.

The endocrine abnormalities accompanying TP include both glucagon and pancreatic polypeptide (PP) deficiency in addition to insulin and thus are considered to be different than conventional type 1 and type 2 DM. TP patients have been thought to be more vulnerable to severe hypoglycemic episodes, tend to be resistant to ketosis, and have a higher plasma level of gluconeogenic precursors, which include lactate and alanine because of glucagon absence [19, 20]. As for pancreatic polypeptide, it has been suggested that it plays a key role in the induction of hepatic sensitivity to insulin and insulin receptor regulation [21, 22]. Following TP, insulin receptors are unregulated peripherally, rendering patients uniquely sensitive to insulin replacement [23], resulting in problematic glycemic control and increased susceptibility to both hyper- and hypoglycemia.

The underlying pancreatic disease may play a role in glycemic control subsequent to TP. Previous studies have shown that patients with chronic pancreatitis tended to have a poorer diabetic outcome [24]. There has been a recent increase in performing TP for malignant diseases of the pancreas [25], benign pancreatic disease [26], patients with genetic abnormalities [27] and premalignant pancreatic disease, mainly IPMN [10–14, 28]. More recent studies looking at outcome after TP show more favorable outcome with both quality of life [5, 29] and in glycemic control [6, 15]. None of these studies focused on IPMN patients.

The improved overall results seen in the past decade may also be multifactorial. Improvements in glucose monitoring systems, insulin delivery systems, and insulin formulations may contribute to superior glycemic control for these patients [30].

Since the first description of IPMN in 1982 by Ohashi et al. [31], IPMN is being increasingly recognized in all parts of the world [32–37]. IPMN has a broad histological spectrum, ranging from minimal mucinous hyperplasia or adenoma to invasive carcinoma [12]. IPMNs are believed to have typical adenoma-carcinoma sequence. The estimated time for this progression is thought to be approximately 5 years [12]. However, it remains a difficult task to determine which IPMN may have malignancy based only on imaging characteristics. This has led to an international consensus on guidelines for management of IPMN including when surgery should be considered [9].

The frequency of malignancy (in situ and invasive) also varies, depending on the type of IPMN. In main duct IPMN, the frequency of malignancy ranges between 60 and 92%, with a mean of 70%. Approximately two-thirds of these malignant neoplasms are invasive [12, 37–44], while in branch duct IPMN, the frequency of malignancy is significantly less, ranging from 6 to 46% [12, 38–44].

One of the main reasons to consider TP in IPMN patients is the increase in survival for those with pancreatic cancer arising in the background of IPMN versus sporadic pancreatic cancer after surgical resection [8]. Another reason is the increased survival in patients with noninvasive IPMN compared to those with invasive IPMN [11, 12, 14, 28, 45, 46], where it can be as low as 24% at 2.5 years [46].

Recurrence of tumor after resection is not uncommon. In a study by Chari et al, 8% of noninvasive IPMNs recurred after partial pancreatectomy compared to none after TP [11]. Interestingly, recurrence was found to be noninvasive in three patients and invasive in two patients [11]. This is in sharp contrast to patients who had invasive IPMN, where recurrence rates after partial pancreatectomy were 67% and after TP were 62% [11]. This emphasizes the need for early detection and aggressive therapy prior to the development of invasive cancer.

Islet cell autotransplants in patients undergoing TP for chronic pancreatitis have shown to have durable function and extended insulin-independence rates, despite a lower beta-cell mass [47]. The fear of infusion of occult carcinoma cells in the islet preparation has limited the use of this procedure for patients with pancreatic adenocarcinoma, although there have been a few published case reports [48, 49]. In one study, islet cell autotransplant was performed in two patients with IPMN, one who underwent TP, and another underwent partial pancreatectomy, in which IPMN was confined to the pancreatic body on imaging, with no evidence of recurrence at one-year followup [50]. IPMN may occur within or away from the intraductal component [51] thus the multicentric nature of IPMN raises a question concerning the suitability of islet cell autotransplantation as an option in the management of these tumors.

The HbA1c levels seen in our post-TP and control patients are comparable to published studies, including those seen in patients after TP [5, 6, 29], in patients with type 1 DM [52, 53], and to type 2 DM patients in the United Kingdom Prospective Diabetes Study (UKPDS) [54].

Hypoglycemia is a feared complication of pancreatogenic diabetes, due to the loss of the counterregulatory mechanism offered by glucagon. The percentage of hypoglycemic episodes in TP patients in our study was similar to that of type 1 DM patients, and none required hospital admission.

 Similar to the study by Jethwa et al. [6], we were unable to find specific reasons for why keeping diabetes under control in this group did not seem to be any more difficult than in patients with autoimmune type 1 diabetes. Better patient understanding of consequences of TP, early education on diabetes (all patients were seen by an endocrinologist immediately following their operation), advances in medical therapy, and blood glucose monitoring could all be contributory factors.

Although use of various types of insulin among patients within both groups made it impossible to make direct comparison, all regimens used were within current guidelines and had the potential to offer excellent glucose control.

Diabetes control is mainly patient driven. Excellent control has been achieved with various insulin regimens, including those used by the patients included in this study.

In addition to improved endocrine control, exocrine insufficiency may be improved by modern pancreatic enzyme formulations. This is important to avoid malabsorption, with its potential negative effects on glycemic control.

This study is not without its limitations. This is a retrospective study conducted at a single center. The length of followup was short; however, this study did not intend to assess long-term glycemic control and complications. Also, hypo- and hyperglycemia were self-reported and therefore, subject to recall bias.

The chief limitation of this study is the small sample size, particularly the small number of TP patients, which resulted in very low power to detect differences between the TP group and the type I DM patients. TP patients were included if they had HbA1c values available at any one of the four time points we considered, and thus our sample size of 14 TP patients was further reduced at each given post-TP time point. Thus, the possibility of a type II error is important to acknowledge.

## 5. Conclusion

These findings suggest that glycemic control following TP for IPMN can be well managed and controlled with a variety of insulin therapy regimens. If these findings are validated in a prospective study that involves a larger number of TP patients, implications are that fear of DM following TP for IPMN should not preclude surgery.

## 6. Study Highlights

1. What is the current knowledge.
2. Glycemic control following total pancreatectomy has been thought to be difficult to manage with potential life-threatening complications.
3. What is new here.
  - Glycemic control following total pancreatectomy for intraductal papillary mucinous neoplasm can be well managed and controlled with a variety of insulin therapy regimens.
  - The mean HbA1c was similar between patient undergoing total pancreatectomy for intraductal papillary mucinous neoplasm and type I DM patients.

## Figures and Tables

**Table 1:** Patient characteristics at baseline.

Variable	TP (*N* = 14)	Type I DM (*N* = 100)	*P* value
Age at baseline (years)	72 (57–78)	52 (21–84)	<0.001
Gender			0.010
Male	2 (14%)	52 (52%)	
Female	12 (86%)	48 (48%)	
Weight at baseline (kg)	60 (50–105)	78 (49–130)	0.028
BMI at baseline	24 (20–36)	26 (20–40)	0.47
Years of education	12 (12–17)	16 (8–18)	0.034
Pancreatic enzyme supplement	13 (93%)	N/A	N/A
Duration of disease (years)	N/A	26 (2–55)	N/A

The sample median (minimum-maximum) is given for numerical variables. Information was unavailable for years of education (*N* = 9). *P*-values result from a Wilcoxon rank sum test or Fisher's exact test. (TP: total pancreatectomy; DM: diabetes mellitus).

**Table 2:** Comparison of HbA1c values after baseline between total pancreatectomy and type I diabetes mellitus patients.

							TP-Type I DM			
Time after baseline		TP-HbA1c (%)			Type I DM-HbA1c (%)		Single variable analysis*		Multivariable analysis^†^	
	Median (min–max)	Mean ± SD	*N*	Median (min–max)	Mean ± SD	*N*	Difference in means (95% CI)	*P* value	Difference in means (95% CI)	*P* value
6 months	7.5 (6.5–8.5)	7.5 ± 0.5	10	7.4 (4.7–11.5)	7.7 ± 1.4	100	−0.14 (−1.00, 0.72)	0.74	−0.05 (−1.05, 0.95)	0.93
12 months	7.3 (5.2–8.4)	7.3 ± 1.0	9	8.0 (5.2–11.8)	8.0 ± 1.2	68	−0.69 (−1.54, 0.15)	0.11	−0.67 (−1.57, 0.23)	0.14
18 months	7.8 (6.9–8.3)	7.7 ± 0.5	7	7.6 (5.3–10.3)	7.6 ± 1.0	65	0.11 (−0.69, 0.90)	0.79	−0.05 (−0.94, 0.84)	0.91
24 months	7.3 (6.6–8.1)	7.3 ± 0.6	6	7.8 (5.4–10.9)	7.8 ± 1.2	58	−0.50 (−1.47, 0.48)	0.31	−0.78 (−1.87, 0.31)	0.16

*Estimates of differences in means and *P* values result from a two-sample *t*-test. ^†^Estimates of differences in means and *P*-values result from linear regression models adjusted for age at baseline, gender, weight, and years of education, all of which differed between TP patients and type I diabetes mellitus patients with a *P*-value of 0.05 or less. (TP: total pancreatectomy; DM: diabetes mellitus).

**Table 3:** Comparison of presence of HbA1c <7% after baseline between total pancreatectomy and type I diabetes mellitus patients.

Time after baseline	TP		Type I DM		TP-Type I DM	
	Fraction (%) with HbA1c <7%	95% CI	Fraction (%) with HbA1c <7%	95% CI	Difference in proportions (95% CI)	*P* value
6 months	1/10 (10%)	0%–45%	33/100 (33%)	24%–43%	−23% (−36%, 9%)	0.17
12 months	3/9 (33%)	7%–70%	14/68 (21%)	12%–32%	13% (−11%, 45%)	0.42
18 months	1/7 (14%)	0%–58%	16/65 (25%)	15%–37%	−10% (−27%, 28%)	1.00
24 months	2/6 (33%)	4%–78%	16/58 (28%)	17%–41%	6% (−21%, 44%)	1.00

*P*-values result from Fisher's exact test. (TP: total pancreatectomy; DM: diabetes mellitus).
